# APX001 Is Effective in the Treatment of Murine Invasive Pulmonary Aspergillosis

**DOI:** 10.1128/AAC.01713-18

**Published:** 2019-01-29

**Authors:** Teclegiorgis Gebremariam, Sondus Alkhazraji, Abdullah Alqarihi, Heewon H. Jeon, Yiyou Gu, Mili Kapoor, Karen J. Shaw, Ashraf S. Ibrahim

**Affiliations:** a Division of Infectious Diseases, Los Angeles Biomedical Research Institute at Harbor-University of California Los Angeles (UCLA) Medical Center and St. John’s Cardiovascular Research Center, Torrance, California, USA; b Amplyx Pharmaceuticals, Inc., San Diego, California, USA; c David Geffen School of Medicine at UCLA, Los Angeles, California, USA

**Keywords:** 1-aminobenzotriazole, APX001, APX001A, *Aspergillus*, Gwt1, IPA, antifungal, infection model

## Abstract

Invasive pulmonary aspergillosis (IPA) due to Aspergillus fumigatus is a serious fungal infection in the immunosuppressed patient population. Despite the introduction of new antifungal agents, mortality rates remain high, and new treatments are needed. The novel antifungal APX001A targets the conserved Gwt1 enzyme required for the localization of glycosylphosphatidylinositol-anchored mannoproteins in fungi. We evaluated the *in vitro* activity of APX001A against A. fumigatus and the *in vivo* activity of its prodrug APX001 in an immunosuppressed mouse model of IPA. APX001A inhibited the growth of A. fumigatus with a minimum effective concentration of 0.03 μg/ml. The use of 50 mg/kg 1-aminobenzotriazole (ABT), a suicide inhibitor of cytochrome P450 enzymes, enhanced APX001A exposures (area under the time-concentration curve [AUC]) 16- to 18-fold and enhanced serum half-life from ∼1 to 9 h, more closely mimicking human pharmacokinetics. We evaluated the efficacy of APX001 (with ABT) in treating murine IPA compared to posaconazole treatment. Treatment of mice with 78 mg/kg once daily (QD), 78 mg/kg twice daily, or 104 mg/kg QD APX001 significantly enhanced the median survival time and prolonged day 21 postinfection overall survival compared to the placebo. Furthermore, administration of APX001 resulted in a significant reduction in lung fungal burden (4.2 to 7.6 log_10_ conidial equivalents/g of tissue) versus the untreated control and resolved the infection, as judged by histopathological examination. The observed survival and tissue clearance were comparable to a clinically relevant posaconazole dose. These results warrant the continued development of APX001 as a broad-spectrum, first-in-class treatment of invasive fungal infections.

## Abstract (precis)

Invasive pulmonary aspergillosis (IPA) due to Aspergillus fumigatus is a serious fungal infection in the immunosuppressed patient population. Despite the introduction of new antifungal agents, mortality rates remain high, and new treatments are needed.

## INTRODUCTION

Invasive pulmonary aspergillosis (IPA) is the leading cause of mold infections in the immunocompromised host. Aspergillus fumigatus is responsible for causing the majority of >200,000 annual cases of invasive aspergillosis worldwide (1). In the last two decades, several antifungal agents have been approved for the prophylaxis or treatment of infections due to *Aspergillus*, including triazoles (voriconazole, posaconazole, and isavuconazole), polyenes, and echinocandins. The guidelines of the Infectious Diseases Society of America (IDSA) recommend triazoles as the preferred agents for first-line therapy (2). Amphotericin B (AmB) and the lipid formulations of AmB are also appropriate treatment options when a triazole cannot be used and/or for salvage therapy. Finally, echinocandins are used for salvage therapy either alone or in combination therapy with triazoles or polyenes (2). Despite the current aggressive antifungal therapy, IPA still results in a high percentage of fatalities (>50%) among severely immunosuppressed patients, such as neutropenic leukemic and transplant patients (3). In addition, significant issues with toxicity and drug-drug interactions may further limit the clinical usefulness of the currently available antifungal agents. Finally, triazole-resistant A. fumigatus are a growing concern due to prolonged drug exposure in patients with chronic pulmonary aspergillosis or due to the environmental exposure of isolates to triazoles used in agriculture (4). Therefore, the development of new therapeutic strategies for invasive aspergillosis is of paramount importance.

APX001 (formerly E1211; 2-amino-3-(3-{4-[(pyridine-2-yloxy)methylbenzyl}-1-2-isoxazol-5-yl)pyridinium-1-yl]methyl hydrogen phosphate) is a first-in-class small molecule antifungal that is currently in clinical development for the treatment of invasive fungal infections (5, 6). APX001 is an *N*-phosphonooxymethyl prodrug which is rapidly and completely metabolized by systemic alkaline phosphatases to the active moiety, APX001A (formerly E1210) (7). APX001A targets the highly conserved fungal enzyme Gwt1, which catalyzes an early step in glycosylphosphatidylinositol anchor biosynthesis (8, 9). Inhibition of Gwt1 prevents the appropriate localization of cell wall mannoproteins, compromising cell wall integrity, biofilm formation, germ tube formation, and fungal growth (10, 11). The closest mammalian ortholog, PIGW, is not sensitive to inhibition by APX001A (10).

APX001A is active against a broad range of pathogenic yeast and molds, including *Candida*, *Coccidioides*, *Cryptococcus*, *Aspergillus*, *Scedosporium*, *Fusarium*, and members of the Mucorales order (12–16). In mouse models of invasive fungal infections, the administration of APX001 (or APX001A) resulted in increased survival and reduced colony counts of fungi in the lungs, kidneys, and brain tissues of infected mice (7, 17–20).

APX001A has been shown to have a half-life in mice of 1.4 to 2.75 h, which is significantly shorter than what has been observed in healthy volunteers during phase 1 clinical studies (2 to 2.5 days) (5, 6, 20). As a result, multiple-daily dosing regimens have been used for evaluation of efficacy in mouse models (7, 19). More recently, 1-aminobenzotriazole (ABT), a well-established time-dependent nonselective suicide inhibitor of cytochrome P450 (CYP) enzymes (21), has been shown to increase the exposure and half-life of APX001A and related compounds in mice when administered 2 h prior to treatment (17, 18). In these studies, once-daily dosing of 100 mg/kg ABT provided APX001A exposure levels previously only observed with very high doses and three times daily (TID) dosing. In this study, we evaluated the pharmacokinetics (PK) of APX001A using a range of ABT doses from 25 to 100 mg/kg with the goal of identifying an optimal dose for *in vivo* efficacy studies. The activity of APX001 was then examined in a well-established neutropenic mouse model of IPA (22). Several endpoints were examined which included survival, histology, and tissue fungal burden, as measured by a quantitative PCR assay that evaluated log_10_ conidial equivalents/g of lung tissue.

## RESULTS

### Effect of ABT on the PK of APX001A.

The PK of APX001A after oral administration of 26 mg/kg of the prodrug APX001 (equivalent to 20 mg/kg of the active moiety APX001A using a conversion factor of 1.3 to account for the methyl phosphate group) were compared with and without the administration of ABT given 2 h prior to APX001 dosing. ABT doses were tested at 25, 50, and 100 mg/kg once daily (QD) and at 50 mg/kg twice daily (BID). Consistent with our previous findings (17), administration of ABT at 100 mg/kg QD resulted in a 15-fold increase in the average APX001A AUC_last_ (area under the plasma concentration-time curve from time zero to time of last measurable concentration) in male CD-1 mice when the prodrug APX001 was dosed at 26 mg/kg (Table 1). Interestingly, this increase in AUC_last_ was maintained when ABT was dosed at 50 mg/kg QD or BID (16.3- or 15-fold versus the no-ABT control, *P* > 0.62 for all ABT comparison regimens) (Table 1), suggesting that this lower dose of ABT is as efficient as the 100-mg/kg ABT dose in enhancing APX001A AUC_last_. In contrast, the 25-mg/kg QD dose of ABT resulted in a lower APX001A AUC value that was statistically significant from the 50-mg/kg QD dose (*P* = 0.02), although a 12.8-fold increase in the AUC value versus the no-ABT control was observed (*P = *0.0002) (Table 1).

**TABLE 1:** Exposures of APX001A following oral dosing of APX001 in the presence or absence of ABT pretreatment

APX001 dose (mg/kg)	ABT dose (mg/kg; dosing frequency)	Avg APX001A AUC^*a*^ (μg·h/ml)	AUC ratio (with ABT/without ABT)
26	None	2.77 ± 0.23	
	100; QD	41.50 ± 8.09	15.0
	None	2.48 ± 1.26	
	25; QD	31.68 ± 3.60	12.8
	50; QD	40.39 ± 1.73	16.3
	50; BID	38.20 ± 7.00	15.4
52	None	5.30 ± 0.98	14.3
	25; QD	52.00 ± 35.46	9.8
	50; QD	94.29 ± 12.43	17.8
	50; BID	92.41 ± 7.70	17.4

a Time course for the Fast PK experiment: 0.083 0.5, 2, 4, 8, and 24 h postdose of 26 mg/kg prodrug (*n* = 3 per time point). AUC is the area under the curve of the analyte, calculated from *T* = 0 to the last measurable concentration.

Since higher APX001 doses could potentially be utilized in efficacy models, it was important to understand the linearity of AUC values while utilizing ABT. Thus, the PK of APX001A after the administration of 52 mg/kg APX001 prodrug (equivalent to 40 mg/kg of the active moiety APX001A) was evaluated in the presence of different doses of ABT. The data in Table 1 show that the administration of ABT at 50 mg/kg BID and 50 mg/kg QD resulted in similar APX001A AUC values (92.41 ± 7.70 and 94.29 ± 12.43, respectively), which translated into a 17.4- to 17.8-fold increase in AUC versus the no-ABT control (5.30 ± 0.98) (*P* < 0.0003). In contrast, the 25-mg/kg QD ABT dose resulted in a lower APX001A AUC value (52.00 ± 35.46), representing a 9.8-fold increase versus the no-ABT control (Table 1).

The AUC values obtained after dosing 52 mg/kg APX001 plus 50 mg/kg ABT (QD or BID) were ∼2-fold higher than the parallel values obtained when 26 mg/kg APX001 was dosed (*P* > 0.14), consistent with dose linearity, at least within that dosing range. We chose to use the lowest, optimal dose of ABT at a 50-mg/kg QD dose in conjunction with the oral administration of APX001 in the subsequent A. fumigatus mouse model experiments.

### ABT has no antifungal effect *in vitro*.

The antimicrobial activity of ABT was evaluated against A. fumigatus using a dilution range of 0.016 to 16 μg/ml. This dilution range was chosen based upon the results of ABT PK in rats, where a single dose of 50 mg/kg ABT resulted in a *C*_max_ of 128 mM or 17 μg/ml (21). No antifungal activity (MIC or the minimum effective concentration [MEC]) was detected using Clinical and Laboratory Standards Institute (CLSI) guideline M38-A2 for molds (23), nor were any changes in cell density observed (data not shown). Standard checkerboard assays (24) were utilized to evaluate potential synergy between ABT and APX001A on A. fumigatus (APX001A ranged from 0.0005 to 0.125 μg/ml; ABT concentrations ranged from 0.016 to 16 μg/ml). Inhibition endpoints using the MEC value were read for assessment of the activity of APX001A against molds. No synergy, additivity, or antagonism was observed. A higher ABT dilution range (0.25 to 250 μg/ml) was also evaluated against A. fumigatus MYA3626 and A. fumigatus AF293. MEC values were determined to be >250 μg/ml, with no evidence of any antifungal effect.

### APX001A has *in vitro* activity against *A. fumigatus* AF293, and the prodrug APX001 protects immunosuppressed mice from IPA.

To evaluate the activity of the prodrug APX001 against *Aspergillus* infections, we first determined the MEC value of the active moiety APX001A against A. fumigatus AF293, a clinical isolate that we have previously used in our inhalational model (22). In a broth microdilution assay, APX001A has an MEC value of 0.03 μg/ml against this strain, as defined by the lowest concentration of drug that leads to small, rounded compact hyphal forms compared to the growth control (23). This clinical isolate is also susceptible to posaconazole with an MIC of 0.5 μg/ml.

Given the impressive *in vitro* activity, we investigated the effect of APX001 in treating immunosuppressed mice with IPA. Cyclophosphamide/cortisone acetate-treated mice were infected (day 0) with A. fumigatus AF293 via inhalation and treated with either APX001 or posaconazole 16 h later. A daily dose of 50 mg/kg QD ABT was administered 2 h prior to APX001 or placebo treatment, from day 1 until day 8.

**FIG 1:**
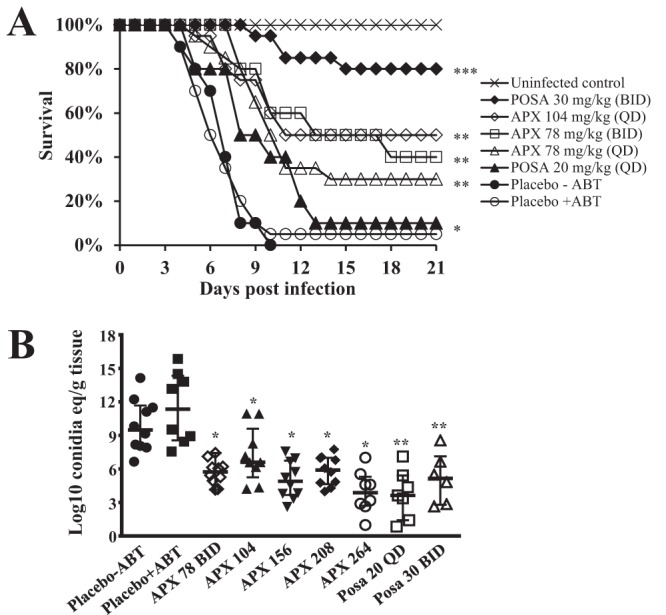
APX001 protects mice from IPA. (A) ICR male mice (*n* = 10 to 20) were infected with an average inoculum of 6.3 × 10^3^ CFU per mouse via inhalation. Treatment was initiated 16 h after infection and continued daily for 7 days. A dose of 50 mg/kg ABT was administered orally 2 h prior to each APX001 dose. ***, *P* < 0.05 versus all other treatments; **, *P* < 0.0005 versus placebo control; *, *P* < 0.05 versus placebo control by log rank test. (B) Lung burdens in mice (6 to 10 per group) were measured at 4 days postinfection. Male ICR mice were infected with 6.7 × 10^3^ CFU via inhalation. Treatment was initiated 16 h postinfection and continued for 4 days. APX001 was administered by oral gavage. ABT was administered orally 2 h prior to each APX001 dose. Mice were sacrificed 8 h after the last dose, and lungs were harvested and processed for tissue fungal burden by qPCR. Fungal burden data (presented as medians ± interquartile ranges) were log_10_ transformed and evaluated using the nonparametric Wilcoxon rank sum test (Prism 5; GraphPad Software, Inc., San Diego, CA). *, *P* < 0.009 versus placebo plus ABT; **, *P* < 0.004 versus placebo without ABT.

Mice treated with either placebo or 50 mg/kg ABT plus placebo demonstrated overlapping survival curves (*P = *0.83), consistent with no effect of ABT treatment alone (Fig. 1A). Treatment of mice with 78 mg/kg APX001 QD or BID (a dose equivalent to 60 mg/kg APX001A) and 104 mg/kg QD (equivalent to 80 mg/kg APX001A) prolonged the median survival time from 6 days for ABT/placebo-treated mice to 10 to 12 days for APX001-treated mice. Importantly, the 21-day survival of mice treated with APX001 at 78 mg/kg QD, 78 mg/kg BID, and 104 mg/kg QD resulted in 30, 40, and 50% survival, respectively, versus 5% for ABT/placebo-treated mice (Fig. 1A). Interestingly, mice treated with posaconazole at 20 mg/kg QD, a dose that is twice as high as the humanized dose required in mice to achieve an AUC consistent with efficacy in the clinic (25), had a median survival time of 8 days and a 21-day survival of just 10%. However, increasing the posaconazole dose to a supratherapeutic level (30 mg/kg BID), a dose which consistently demonstrates efficacy in this model, had a superior activity to all treatment arms with a median survival time of >21 days and an overall 21-day survival of 80% (Fig. 1A).

### APX001 reduces lung fungal burden, as assessed by log_10_ conidial equivalents/g of lung tissue reduction and histological observations.

Because APX001 increased the survival rates of immunosuppressed mice with IPA, the effect of the drug treatment on tissue fungal burden in the lungs was determined in a new set of experiments which used quantitative PCR (qPCR) to evaluate log_10_ conidial equivalents/g of tissue. Mice were infected and treated as in the survival studies and then sacrificed on day 3 (8 h after the last treatment), and their lungs (primary target organ) (22) were harvested and processed for the determination of tissue fungal burden by qPCR (26). Concordant with the *in vitro* results, treating mice with placebo plus ABT did not result in a significant difference in log_10_ conidial equivalents/g of lung tissue versus the placebo without ABT control (Fig. 1B), thereby confirming the lack of *in vivo* activity of ABT against A. fumigatus . In contrast, treating mice with the 104 mg/kg QD of APX001 plus ABT resulted in a 4.75-log_10_ conidial equivalents/g decrease in lung fungal burden versus placebo/ABT-treated mice (Fig. 1B).

In this experiment, we also evaluated higher doses of APX001 (156, 208, and 264 mg/kg QD equivalent to 120, 160, and 200 mg/kg of APX001A, respectively) plus ABT on lung fungal burden versus the placebo+ABT control to investigate whether the reduction in fungal burden is dose dependent. Indeed, these elevated APX001 doses resulted in larger reductions in fungal burden of at least 5.65 to 7.64 log_10_ conidial equivalents/g of lung tissue, which was consistent with dose-dependent activity (Fig. 1B) (*P <* 0.05 for APX001-264 versus APX001-104 or APX001-208). However, such high doses of APX001A appear to be toxic in immunocompromised mice when dosed over a long period of time, since we found them to be associated with a dose-dependent loss of activity in survival studies. Specifically, a dose of 156 mg/kg APX001A resulted in minimal enhancement of the median survival time of 8 days and a 10% overall survival versus a 7-day median survival time and a 0% overall survival for placebo-treated mice (*P = *0.03). Furthermore, doses higher than 156 mg/kg had no benefit in enhancing the survival of mice (data not shown) despite the reduction achieved in CFU (Fig. 1B). Finally, posaconazole treatment resulted in a similar reduction in log_10_ conidial equivalents/g of lung tissue compared to APX001-264 mg/kg (Fig. 1B).

**FIG 2:**
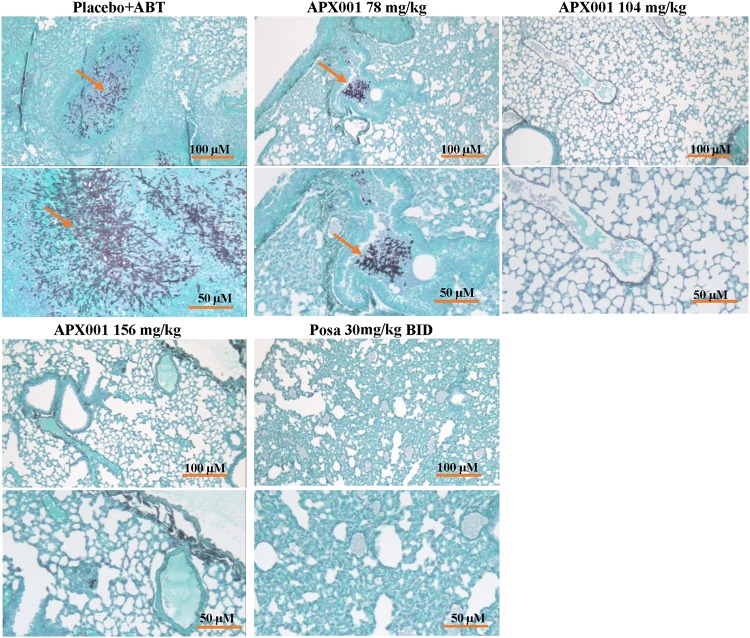
Histological examination of lungs harvested from mice treated with placebo, APX001, or posaconazole. Mice were infected and treated as in Fig. 1B. Harvested, fixed lungs were stained with GMS prior to microscopic examination. Notice the focal fungal pneumonia (indicated by the abscesses in the placebo mice with elongated intact hyphae) and tissue edema versus a smaller abscess in the 78-mg/kg APX001 dose image with fragmented fungal hyphae and less tissue edema (arrows). Treatment with higher doses of APX001 or posaconazole resulted in normal lung architecture with no signs of fungal pneumonia.

Histopathological examination of lungs harvested from mice on day 4 postinfection showed that ABT/placebo-treated mice had multiple large parenchymal abscesses of A. fumigatus hyphae surrounded by phagocytes with signs of necrotizing fungal pneumonia and a substantial degree of tissue edema (Fig. 2). In contrast, lungs harvested from mice treated with APX001 at 78 mg/kg QD had fewer parenchymal abscesses of mainly fragmented A. fumigatus hyphae with less hemorrhage and tissue edema. Furthermore, lungs harvested from mice treated with 104 or 156 mg/kg QD APX001 or with posaconazole (30 mg/kg, BID) showed no fungal abscesses and normal tissue architecture (Fig. 2), which is consistent with dose-dependent activity. These results confirm the similar efficacies of APX001 and posaconazole in this IPA murine model.

## DISCUSSION

The prodrug APX001 is a first-in-class, intravenous and orally available broad-spectrum antifungal agent in clinical development for the treatment of life-threatening invasive fungal infections. Previous studies have shown that APX001A, the active moiety of APX001, has *in vitro* activity against a variety of yeast and molds. In two studies, the MEC_90_ values for A. fumigatus have been reported to be 0.13 μg/ml (13) or 0.06 μg/ml (15) using CLSI methodology. Similar or lower MEC_90_ values were seen with other species of *Aspergillus* (A. flavus, 0.03 μg/ml; A. niger, 0.015 μg/ml; and A. terreus, 0.06 μg/ml), and activity was also observed against AmB-resistant strains of A. terreus and itraconazole-resistant strains of A. fumigatus (15). Consistent with these reported values, the MEC of APX001A against A. fumigatus AF293 was found to be 0.03 μg/ml. These values compare favorably to other drugs in clinical use for IPA. For example, a recent study reported the activities of isavuconazole, itraconazole, posaconazole, and voriconazole against A. fumigatus to range between 0.12 and 32 μg/ml, 0.12 and 32 μg/ml, 0.008 and 4 μg/ml, and 0.12 and 32 μg/ml, respectively (27).

The pan-CYP450 inhibitor ABT has been shown to dramatically increase the exposure of APX001A in several animal models when administered once daily 2 h prior to the administration of the prodrug APX001 (17, 18). Since efficacy models can require dosing for 7 days or longer, the ability to maintain good drug exposures by administration of ABT over the treatment period is important. In these studies, significant reductions in the log_10_ CFU/g of tissue were observed in kidneys (C. albicans and C. glabrata) and lungs and brain (C. neoformans) due to the >9-fold increase in APX001A exposures that were observed with ABT predosing, despite QD dosing of APX001. Similarly, we show that the ABT increased the AUC_last_ values of APX001A, prolonged its half-life in mice, and potentiated a significant activity in protecting against murine IPA.

A previous study had shown that APX001 was effective in IPA models of A. fumigatus and A. flavus (28). In that model, mice were dosed intraperitoneally with 52 mg/kg APX001 (previously E1211) TID for 5 days to achieve 100% survival. The corresponding daily AUC values would be approximately 26 to 46 μg ⋅ h/ml (18; data not shown). One caveat to the previous study is that the mice were immunosuppressed with 200 mg/kg of 5-fluorouracil administered subcutaneously 5 to 6 days prior to infection, with the nadir of the neutrophil counts occurring approximately on the day of infection (29), indicating that the neutrophils were recovering during the early course of the infection. The IPA model in the present study utilized more severely immunocompromised mice in which the cyclophosphamide/cortisone acetate treatment results in pancytopenia for at least 9 days from the first administered dose (22). Thus, using ABT plus 104 mg/kg APX001 resulted in approximately 4- to 11-fold-higher AUC_last_ values (Table 1 and data not shown), exposures that were necessary for achieving significant efficacy in this severely immunocompromised mouse model.

The mouse survival studies clearly demonstrate a survival benefit, tissue fungal burden reduction, and histological clearance of infection when mice were treated with APX001. This activity of APX001 treatment of IPA was similar to posaconazole treatment at 20 mg/kg QD, a dose that is twice as high as the 10-mg/kg posaconazole dose required in mice to achieve an AUC consistent with efficacy in the clinic (25). The 20-mg/kg dose was also shown to produce maximal suppression of the galactomannan biomarker using the same murine IPA model (30). A survival benefit of posaconazole treatment over APX001 was seen only when a very high dose of posaconazole (60 mg/kg/day administered as 30 mg/kg BID) was used.

APX001 phase 1 single and multiple ascending dose studies were recently completed. APX001 was very well tolerated across all administered doses of oral or intravenous formulations. In addition, the favorable PK allows a single daily administration with the ability to switch between oral and intravenous formulations (5, 6). Given the broad-spectrum activity of APX001A, including its activity against azole- and AmB-resistant isolates of *Aspergillus* species, the comparable activity of APX001 to posaconazole in our murine IPA model, and the good safety profile and favorable PK in humans, further investigations into the development of this first-in-class agent are highly warranted.

## MATERIALS AND METHODS

### Microorganisms.

A. fumigatus strains AF293 and MYA3626 were used in this study and routinely grown on Sabouraud dextrose agar plates for 10 to 15 days until confluent at 37°C. Both strains were used for the *in vitro* studies, while only strain AF293 was used for *in vivo* testing. Conidia were collected by flooding the plates with sterile phosphate-buffered saline containing 0.2% (vol/vol) Tween 80. The conidia were concentrated by centrifugation, washed in the same buffer, diluted, and counted using a hemocytometer.

### Antifungal agents.

For pharmacokinetic and efficacy studies, the prodrug APX001 (Amplyx Pharmaceuticals) were used. APX001, the *N*-phosphonooxymethyl prodrug, is soluble in water. Final prodrug solutions were in 5% dextrose and dosed orally per gram of mouse body daily weight basis. A 5-mg/ml solution of ABT (Fisher Scientific, Hampton, NH) in water was administered orally 2 h prior to infection as 5, 10, or 20 μl per gram of mouse body weight, resulting in doses of 25, 50, or 100 mg/kg, respectively. Posaconazole (Merck & Co., Inc., Rahway, NJ) was purchased as an oral suspension (200 mg/5 ml) and kept at room temperature.

### Antifungal susceptibility testing.

To establish the antimicrobial activity of APX001A analogs, broth microdilution susceptibility testing was performed according to CLSI guideline M38-A2 for molds (23). APX001A were first diluted in dimethyl sulfoxide (DMSO) to obtain intermediate dilutions. These were further diluted in microtiter plates to obtain a final concentration of 0.002 to 2 μg/ml. The, 1 μl of DMSO was added to “no drug” control wells. The solutions were mixed on a plate shaker for 10 min, and plates were incubated at 35°C for 40 to 48 h. The minimum concentration that led to shortening of hyphae compared to hyphal growth in DMSO control wells was determined as the MEC for A. fumigatus (as read for echinocandins). Similar methods were used to determine the effect of ABT on the growth of A. fumigatus, with the exception that DMSO was not used because ABT is a water-soluble molecule. The range of ABT concentrations was 0.016 to 16 μg/ml in one study and 0.25 to 250 μg/ml in a follow-up study. The use of the MIC and MEC endpoints for APX001A (formerly E1210) against yeasts and molds, respectively, has been described previously (13–16). Standard checkerboard assays (24) were utilized to evaluate synergy between ABT and APX001A on A. fumigatus MYA3626 (APX001A concentrations ranged from 0.0005 to 0.125 μg/ml; ABT concentrations ranged from 0.016 to 16 μg/ml). Inhibition endpoints for the synergy assay were read using the MEC value, as read for assessment of the activity of APX001A against molds.

### Pharmacokinetic analysis.

A single-dose rapid screening methodology (Fast PK) was used to evaluate the PK in healthy immunocompetent male CD-1 mice following oral dosing of 26 or 52 mg/kg with the prodrug APX001 (5% glucose, NaOH [pH 7.1]; Absorption Systems, San Diego, CA). Mice (*n* = 3) received a single oral dose of ABT at 25, 50, or 100 mg/kg 2 h prior to prodrug dosing. Blood samples (25 μl) were sequentially collected from the tail vein 0.083, 0.5, 2, 4, 8, and 24 h postdose and mixed with 25 μl of heparinized water. Hemolyzed blood samples were manually extracted by protein precipitation using acetonitrile in 96-well plates. Samples were extracted and analyzed by liquid chromatography-tandem mass spectrometry to determine average blood concentrations (Table 1). Samples that were below the limit of quantification (1 ng/ml) were not used in the calculation of averages. Pharmacokinetic parameters were calculated from the time course of the blood concentrations using Phoenix WinNonlin (v7.0) software using a noncompartmental model. The AUC was calculated using the linear trapezoidal rule with calculation to the last quantifiable data point. Samples below the limit of quantitation (1 ng/ml) were treated as zero for pharmacokinetic data analysis. *P* values were determined by a paired *t* test (two-tailed distribution).

After oral administration of APX001 prodrug at 52 mg/kg (ABT at 25 mg/kg), one mouse was found dead prior to the blood sample collection at 8 h; the cause of death was unknown. A second mouse was found extremely lethargic at 8 h postdosing; therefore, the animal was terminated after the blood sample collection at 8 h. No other adverse reactions were observed after the oral administration of APX001 prodrug in male CD-1 mice during this study.

### IPA model.

The IPA model was performed as previously described (22). Briefly, immunosuppressed mice were challenged with A. fumigatus in an inhalation chamber by aerosolizing 12 ml of a 1 × 10^9^ ml suspension of conidia with a small particle nebulizer driven by compressed air (22). A standard exposure time of 1 h was used for all experiments. Immediately after infection, a subset of the mice was sacrificed, and the lungs were removed for quantitative culture. Mice were rendered neutropenic using a regimen of 200 mg/kg cyclophosphamide and 500 mg/kg cortisone acetate 2 days before and on day 3 relative to infection. To prevent bacterial infection, mice were given Baytril (50 μg/ml of enrofloxacin; Bayer) added to the drinking water from day –3 to day 0. Ceftazidime (5 μg/dose/0.2 ml) replaced Baytril treatment on day 0 and was administered daily by subcutaneous injection from day 0 until day 8. We administered 50 mg/kg ABT orally 2 h before the administration of APX001 for 7 days. Posaconazole (20 mg/kg QD or 30 mg/kg BID) was administered orally for 7 days. Survival was monitored through day 21. Mice were given free access to water and standard laboratory diet. All drug treatments were initiated 16 h postinfection and continued for 8 consecutive days given by oral gavage.

For tissue fungal burden and histopathological examination, mice were infected as described above, and treatment started 16 h postinfection and continued until day 3 (3 days of treatment) prior to sacrificing the mice 8 h after the last treatment. The lungs were harvested and processed for tissue fungal burden determination using qPCR with 18S rRNA gene as previously described (26). Lungs harvested from mice sacrificed at the same time as the tissue fungal burden studies were also processed for histopathological examination. Briefly, tissues were fixed in 10% zinc-buffered formalin, paraffin embedded, sectioned, and stained with Grocott’s methenamine silver (GMS) stain for microscopic examination.

All animal related study procedures were compliant with the Animal Welfare Act, the *Guide for the Care and Use of Laboratory Animals*, and the Office of Laboratory Animal Welfare and were conducted under an IACUC approved protocol by Los Angeles Biomedical Research Institute at Harbor-UCLA Medical Center.

### Statistical analysis.

A nonparametric log-rank test was used to determine differences in survival times. Differences in tissue fungal burdens were compared by the nonparametric Wilcoxon rank sum test for multiple comparisons. A *P* value of <0.05 was considered significant.
